# Effectiveness of Aqueous Extract of Marine Baitworm *Marphysa moribidii* Idris, Hutchings and Arshad, 2014 (Annelida, Polychaeta), on Acute Wound Healing Using Sprague Dawley Rats

**DOI:** 10.1155/2020/1408926

**Published:** 2020-11-25

**Authors:** Hannah Syahirah Rapi, Nor ‘Awatif Che Soh, Nurul Shahirah Mohd Azam, M. Maulidiani, Suvik Assaw, Mohd Nizam Haron, Abdul Manaf Ali, Izwandy Idris, Wan Iryani Wan Ismail

**Affiliations:** ^1^Cell Signaling and Biotechnology Research Group (CeSBTech), Faculty of Science and Marine Environment, Universiti Malaysia Terengganu, 21030 Kuala Nerus, Terengganu, Malaysia; ^2^Faculty of Science and Marine Environment, Universiti Malaysia Terengganu, 21030 Kuala Nerus, Terengganu, Malaysia; ^3^School of Animal Science, Faculty of Bioresources and Food Industry, Universiti Sultan Zainal Abidin, 22200 Besut, Terengganu, Malaysia; ^4^School of Agriculture Science and Biotechnology, Faculty of Bioresources and Food Industry, Universiti Sultan Zainal Abidin, 22200 Besut, Terengganu, Malaysia; ^5^South China Sea Repository and Reference Centre, Institute of Oceanography and Environment (INOS), Universiti Malaysia Terengganu, 21030 Kuala Nerus, Terengganu, Malaysia; ^6^Biological Security and Sustainability (BioSeS) Research Group, Faculty of Science and Marine Environment, Universiti Malaysia Terengganu, 21030 Kuala Nerus, Terengganu, Malaysia; ^7^Institute of Tropical Aquaculture and Fisheries (Akuatrop), Universiti Malaysia Terengganu, 21030 Kuala Nerus, Terengganu, Malaysia

## Abstract

Wound healing is a well-coordinated process that restores skin integrity upon injury. However, some wound treatment poses harmful effects on the skin, which delay the normal wound healing process. *Marphysa moribidii*, a marine baitworm or polychaete, represents unique ability to regenerate posterior segment after injury, which may be beneficial in the wound healing treatment. The effectiveness of the polychaete as wound healing treatment was discovered through skin irritation, microbial testing, animal wound model, and chemical identifications. Three polychaete extracts (PE) emulsifying ointment (0.1%, 0.5%, and 1.0%) were topically applied to the full thickness wound model once daily for 14 days. Interestingly, PE 1.0% revealed the most rapid wound healing effects as compared to other treatments, including gamat (sea cucumber) oil (15% w/v) and acriflavine (0.1% w/v). Histopathological analysis using Masson's trichrome staining further confirms that PE treated wound exhibited minimal scar, high collagen deposition, and the emergence of neovascularisation. The extract also displayed a minimum inhibitory concentration (MIC) of 0.4 g/ml against *Escherichia coli* and absence of skin irritation, infectious bacteria, and heavy metals from the extract. Moreover, chemical compounds such as alkaloid, flavonoid, amino acids, and organic acid were detected in *M. moribidii* extracts, which could contribute to wound healing activity. In conclusion, this study further justifies the beneficial use of polychaete in treating wound healing and could be developed as a novel bioactive agent in nutraceuticals and pharmaceutical drugs.

## 1. Introduction

Skin acts as physical barriers protecting and sealing the body from the external environment and pathogenic substances. Once this first line of defence is broken, a wound is created, which can cause inflammation and bacterial infections on the intact skin. Generally, a wound can be divided into acute and chronic, which much rely on treatment and management. An acute wound that usually heals for two to three weeks includes surgical wounds, traumatic wounds, cuts, and burns. In contrast, chronic wound such as a fungating lesion, pressure ulcer, leg ulcer, and diabetic foot ulcer usually take longer to heal, resulting in incomplete healing [1]. An acute wound may develop into a chronic wound if the healing process fails to progress in time. This makes the injury even worse and challenging to manage. In severe cases, the chronic wound caused death and morbidity. Thus, treating an acute wound is crucial to achieving rapid wound closure, thus preventing the symptom of a nonhealing wound. The wound healing process occurs continuously in a highly integrated and overlapping phase, divided into three: (1) inflammation and homeostasis, (2) proliferation, and finally (3) tissue remodelling and maturation.

Generally, delayed wound closure and impaired healing are caused by a failure in some stages of the wound healing events. Moreover, the presence of skin-related bacteria such as *Staphylococcus aureus*, *S. epidermidis*, *Escherichia coli*, *Pseudomonas aeruginosa*, and *Klebsiella pneumoniae* poses a high risk for wound infection which can slow down the healing process [2]. Meanwhile, some of the market's synthetic wound medicine has been reported with safety issues, an allergic reaction, and skin irritation. These problems eventually prolong the healing process, resulting in an imperfect wound aesthetic. The wound had been a severe problem and a cost-associated issue in public health. In 2018, in an analysis performed by Medicare beneficiaries, the United Kingdom alone had about 8.2 million people suffering from wound problems, which include standard and infected wounds [3]. The analysis also revealed that wound accounts for about $28.1 billion to $96.8 for both acute and chronic treatment. According to Jarić et al. [4], the development of natural materials as potential therapeutic drugs will be useful for effective wound healing remedies with low cost and less adverse events.

The use of annelids such as earthworm (class: Oligochaeta) and leech (class: Hirudinea) have been reported in the literature for their wound healing ability. However, there is no scientific study on polychaete (class Polychaeta) as a potential wound healing agent, despite its ability to self-regenerate. *Marphysa moribidii*, a soft-bodied marine polychaete, is found abundantly in the mangrove forest of Peninsular Malaysia. The species is currently mainly used as baitworm for recreational and artisanal fisheries. Nevertheless, the ability for unique self-regeneration after an injury that caused losing the posterior region [5] probably indicates that *M. moribidii* is composed of various compounds that would help in wound healing. Meanwhile, due to overexploitation of gamat (sea cucumber; Holothuroidea) as successful traditional wound medicine in Southeast Asia, the gamat's population is declining over the years, and a number of gamat species have been listed as threatened with extinction by the International Union for Conservation of Nature (IUCN) Red List. Therefore, the increasing financial burden, drawbacks, and depletion in the current treatment urges the development of wound remedy from new resources that posed effective healing, yet are more cost-effective, safe, and user-friendly. Thus, the present study aims to discover the potential of marine worm *M. moribidii* as an alternative wound healing medicine.

## 2. Materials and Methods

### 2.1. Samples Collection and Storage

Samples of *M. moribidii* were collected during spring low tide from the mangrove *Rhizophora* spp. zonation of the west coast of Peninsular Malaysia. The polychaetes were removed gently from mangrove roots and placed in a container with natural sediment before being transported back to the laboratory. In the laboratory, *M. moribidii* was cleaned using distilled water and stored in −80°C freezer for further use [6].

### 2.2. Preparation of Polychaete Crude Extract

The preparation of polychaetes aqueous extracts was done according to the methods suggested by Mazliadiyana et al. with modifications [7]. Samples were thawed, weighed, and cut into smaller pieces and pulverized and homogenized without water using mortar and pastel. Then, distilled water was added into the sample in 1 : 10 ratio and soaked overnight. The filtered water was collected in flask A, wherein the homogenized *M. moribidii* was further soaked for the next 4 h, followed by centrifugation at 3,000 rpm for 20 min. Then, the resultant supernatant was collected in flask B. Next, water in both flask A and B was mixed and stored at −80°C freezer. After 24 h, the sample was lyophilized in Freeze Dry System/Freezone 4.5 (Canada) for 72 h.

### 2.3. Preparation of *Marphysa moribidii* Ointment

Aqueous extract polychaete in ointment form was prepared in different concentrations (0.1%, 0.5%, and 1.0% (w/w)) by emulsifying the freeze-dried sample of *M. moribidii* polychaete extract (PE) in cetomacrogol emulsifying ointment. Then, the aqueous extract polychaete ointments were stored in a small container at room temperature.

### 2.4. Animals and Experimental Design

Thirty-six male Sprague Dawley (SD) rats (200–260 g) were housed individually in a cage under 12 h light/dark cycle. Throughout the experimental period, food pellet and water were given ad libitum. The study protocol was approved by the Research Ethical Committee, Universiti Malaysia Terengganu (UMT/JKEPHT/2017/10). At least five animals were used for each treatment (*n* = 5). There were altogether seven groups of treatment that were tested on SD rats, namely, three concentrations of aqueous emulsifying ointment of *M. moribidii* polychaete extract (PE; w/w) (PE 0.1%, PE 0.5%, and PE 1.0%), two positive controls of commercialized wound healing products (w/v) (15.0% gamat oil (PCG)) and 1.0% acriflavine (PCA), and two negative controls (cetomacrogol emulsifying ointment (NC) and no treatment applied (NO) as the untreated group).

### 2.5. Effectiveness of Marphysa moribidii Ointment

#### 2.5.1. In Vivo Acute Wound Healing Model

To evaluate the potential of *M. moribidii* aqueous extract emulsifying ointment as a wound healing treatment, the full thickness wound was created on the rat's back using 8 mm disposable sterile biopsy punch. Rats were anaesthetized using 100% diethyl ether and hair-shaved and the skin was sterilized using 10% iodine. Then, all treatments were topically applied once daily on the rats' wound for 14 days except for the NO group. Gross observation on the wound healing process was done on days 0, 3, 7, 11, and 14 after wounding. To determine the wound contraction and epithelialization period, the wound area that remains exposed was measured and traced using a transparent film. Then, the traced wound margin was placed on a graph paper (mm^2^), and the number of squares covered the outlined area was counted, which reflects the wound area [8]. The healing rate was expressed as the percentage of wound contraction and calculated as follows [8]:

(1) $$\begin{array}{c} \text{wound contraction }\left(\text{\%}\right)\;=\frac{\text{Wound area on day 0}-\text{Area of inner wound on day }X}{\text{Wound area of day 0}\times 100}. \end{array}$$

Rats skin (approximately 1 × 1 cm^2^) with wounded area or scar was excised on day 14 and fixed in specimen collection bottle containing 10% buffered formalin for further histological examination.

#### 2.5.2. Rat's Behaviour

Behavioural categories such as resting, sleeping, and cage exploration (walking and climbing) were accessed according to Hashim et al. [9] and Vukojević et al. [10]. The observation was done in the morning for a fixed duration of 30 minutes while handling the experiment on days 0, 3, 7, 11, and 14. Rat's behaviours that include pain responses such as scratching and licking in their wound area were also recorded.

#### 2.5.3. Hematoxylin and Eosin (H&E) and Masson's Trichome (MT) Staining

The fixed tissue sample was subjected to a series of increasing ethanol concentrations (70%, 90%, 95%, and 100%) using tissue processor, hardened into paraffin block, cut into 7 *μ*m tissue ribbon, and secured on a glass slide. Then, the sectioned tissue sample was stained using H&E and MT staining, following the method suggested by Suvik and Effendy with some modifications [11]. Slide deparaffinization was done in xylene, while other steps include soaking in a few types of chemical solutions, staining, gentle wash under running tap water, and drying. Before sample observation, the slide was mounted with DPX and cover using coverslip. Skin reepithelialization, scar formation, angiogenesis, and collagen arrangement from the fixed tissue sample were observed using a light microscope.

### 2.6. Safety Evaluation of *Marphysa moribidii* Ointment

#### 2.6.1. Skin Irritation Test

The safety evaluation of *M. moribidii* aqueous extract emulsifying ointment was done on SD rats' skin via skin irritation test as per OECD 404 guidelines [12]. About 0.5 g samples of NC, PE 0.1%, PE 0.5%, and PE 1.0% were applied uniformly on the rats' skin (approximately 6 cm^2^) and then covered with gauze patch. At 1, 24, 48, and 72 h, skin reactions such as oedema and erythema were examined. At the same time, scoring was tabulated according to OECD guidelines, where 0, 1, 2, 3, and 4 indicated no, very slight, moderate, and severe formation of oedema/erythema, respectively.

#### 2.6.2. Microbial Contamination Test (MCT)

The best concentration of *M. moribidii* aqueous extract emulsifying ointment that effectively works on the rats' wound was evaluated for MCT [13]. The test reveals the value for the total aerobic microbial count (TAMC), total mould and yeast count (TYMC), and the presence of specific bacteria. The acceptance TAMC and TYMC values of a nonsterile product for cutaneous use are summarized in Supplementary Table S1. About 1 g of polychaete ointment was dispersed in 4 ml sterile Ringer solution containing 0.25% tween 80 performed in serial dilution (1 : 10). Then, 1 ml of the mixture was plated on tryptic soy agar (incubated at 30° for five days) and Sabouraud dextrose agar (incubated at 25°C for five to seven days) for determination of TAMC and TYMC, respectively. For determination of *P. aeruginosa* and *S. aureus* absence, 10 ml of the polychaete ointment (PE 1.0%) was mixed with 90 ml buffered NaCl peptone; then 10 ml of the solution mixture was added into 100 ml tryptic soy broth and incubated at 35°C. After 24 h, the mixture was subcultured on cetrimide agar for *P. aeruginosa* and mannitol salt agar for *S. aureus* detection. Automatic Vitek 2 Compact system (bioMerieux, France) was used for bacterial identification.

#### 2.6.3. Heavy Metals Detection

Heavy metals, including arsenic (As), cadmium (Cd), lead (Pb), and mercury (Hg), were analyzed by MyCO2 Laboratory Shah Alam, Selangor, Malaysia, following the analytical method adopted from Association of Official Analytical Chemists (AOAC).

### 2.7. Antibacterial Activity

#### 2.7.1. Bacterial Preparation

*Pseudomonas aeruginosa*, *S. aureus*, *S. epidermidis*, *E. coli*, and *K. pneumonia* species were obtained from Microbiology Laboratory of Faculty of Science and Marine Environment, Universiti Malaysia Terengganu (UMT). These bacteria were chosen due to their direct relationship on human skin and suspended in the environment. All bacteria were cultured on nutrient agar (NA) at 37°C 18 to 24 h to get single colonies. Also, bacterial inoculum for minimum inhibitory concentration (MIC) assay was prepared through direct colony suspension as recommended by the Clinical Laboratory Standards Institute (CLSI). Four to five bacterial colonies from each strain were inoculated into 4 ml of 0.9% sterile saline. Then, the inoculum was measured using UV-VIS spectrophotometer (UV 1800, Shimadzu, Japan) at an optical density of 625 nm (OD_625_) and adjusted to obtain a range of 0.08 to 0.12 nm, which is equivalent to a 0.5 McFarland standard. Then, the inoculum was further diluted according to 1 : 150, to obtain an approximate amount of 5 × 10^5^ CFU/mL bacterial count and ready for MIC assay.

#### 2.7.2. Minimum Inhibitory Concentration and Minimal Bacterial Concentration Assays

Aqueous *M. moribidii* extract was studied for its antibacterial activity against all five types of bacteria. These were done via MIC, followed by minimal bactericidal concentration (MBC) assay. For MIC assay, all bacterial strains were subjected to the broth dilution method, as suggested by Balouiri et al. [14]. Plates were prepared in triplicate and wrapped using aluminium foil before incubation at 37°C for 18 to 24 h. The detailed procedure is summarized in Supplementary Figure S1. For bacterial growth indication, 30 *μ*l of 5 mg/ml tetrazolium salts, 3-(4,5-dimethylthiazol-2-yl)-2,5-diphenyltetrazolium bromide (MTT) solution was added into each well and further incubated for 1 h. MIC endpoint was determined macroscopically by referring to the lowest well concentration that displayed no bacterial growth, whereas MBC assay was done by streaking the bacterial mixture from well at minimum inhibitory concentration (MIC) endpoint on the Mueller Hinton agar (MHA). Then the plate was incubated at 37°C for 18 h for observation of any bacterial growth. The MBC was determined when there was no visible growth of a bacterial colony on MHA media. MBC to MIC ratio was calculated to determine either the crude polychaete extract is bacteriostatic or bactericidal.

### 2.8. Detection of Chemical Compounds

#### 2.8.1. Qualitative Chemical Analysis

The crude extract of *M. moribidii* was assessed to evaluate the presence of a respective class of chemical and metabolites using methods previously described by Gul et al. and Auwal et al. [15, 16].

*(1) Test for Alkaloid*. About 5 ml of 10% ammonia chloroform solution (pH 9) was added into 2 g of *M. moribidii* crude extract. Then, the extract was filtered using cotton plugs into a clean test tube. Then, about 1 ml of 2N H_2_SO_4_ was added into the filtrate and vigorously shook for 15 s. The mixture was left for a while until two liquid phases were formed. Next, 1 ml of acetate extract was removed into another test tube, and about two to three drops of Mayer reagent were added into it. The formation of a white precipitate indicates the presence of alkaloids.

*(2) Test for Flavonoid*. The presence of flavonoids in the sample was analyzed using Shinoda test. Aqueous crude polychaete extract was mixed with few pieces of magnesium ribbon, which had been previously dissolved in methanol. Then, about two to three drops of concentrated HCl were added into the test tube. After a few minutes, red spot formation indicates the presence of flavonoids.

*(3) Test for Saponin*. About 2 g of *M. moribidii* extract was mixed thoroughly in a test tube with 20 ml of distilled water. The mixture was heated on a water steamer and filtered. Then, 10 ml of filtrate was transferred into a beaker and mixed vigorously with 5 ml of distilled water. Next, about three drops of olive oil were added and mixed vigorously. The formation of emulsions indicated the presence of saponin.

*(4) Test for Terpenoid*. The mixture of 5 ml of aqueous polychaete extract and 2 ml chloroform was evaporated in a water bath. Then, the mixture was boiled with 3 ml of concentrated H_2_SO_4_. Terpenoids were indicated by reddish-brown formation.

#### 2.8.2. ^1^H NMR Analysis

^1^H NMR analysis of *M. moribidii* extract was performed on a 400 MHz NMR machine (Bruker Avance, Germany) with parameter settings as follows: acquisition time of 4.29 min (64 scans), the temperature of 26°C, relaxation delay (RD) of 2.0 s, and pulse width (PW) of 21.0 *μ*s (90°). About 10 mg crude extract of *M. moribidii* was added in a vial and mixed with 0.6 ml deuterated DMSO containing 0.03% of trimethylsilane (TMS). The mixture was then vortexed and centrifuged at 13,000 rpm for 10 minutes. The supernatant was then transferred into the NMR tube and subjected to ^1^H NMR analysis. The NMR spectrum was processed and analyzed using MestReNova 8.1 software (Mestrelab Research S.L., Spain) and Chenomx NMR Suite Profiler (Chenomx Inc., Canada). The identification of metabolites was aided by comparing its ^1^H NMR characteristics with an available online database (https://hmdb.ca/) and published literature.

### 2.9. Statistical Analysis

Data for WHP was expressed as mean ± standard error mean (SEM) (*n* = 5). To analyze and compare the significant difference between the treatment groups, one-way analysis of variance (ANOVA) and post hoc comparison using Tukey's honest significant difference (HSD) test were conducted using SPSS (version 22.0) software. A value of *p* < 0.05 was considered statistically significant.

## 3. Results and Discussion

### 3.1. Wound Contraction Analysis

Generally, delayed wound closure is caused by a failure in some phases of the wound healing events or foreign tissues that are not destroyed. In this study, the gross observation on the wound healing process demonstrated that untreated wound in the NO group exhibited a slow healing process with incomplete wound closure at day 7 after wounding which manifested with the formation of granulation tissues, cardinal signs of inflammation such as reddishness, and also minor bleeding (Supplementary Figure S2). Moreover, at day 14 after wounding, NO group also produced scar and keloid tissue. Meanwhile, similar wound healing pattern was observed in 0.1% and 0.5% polychaete ointment (PE 0.1% and PE 0.5%) where the granulation tissue proliferates successfully and then finally induced wound reepithelialization as shown on day 7. Nevertheless, both wound sizes are quite large when compared to PE 1.0% on the same day. Days 11 and 14 demonstrated an ongoing collagenous scar, which rebuilt at the healing site without any pus, bleeding sign, and microbial infections. Interestingly, wound treated with polychaete extract PE 1.0% showed the most visible faster wound contraction with less scar formation, suggesting an active proliferation phase and resolution of inflammatory phase that occurred on day 7 when compared to other treatment groups. The wound reduction is caused by the action of myofibroblast that proliferates and migrates during a continuous proliferative phase [17].

At day 11, no oedema, keloid, or hypertrophic scar formation was observed in all polychaete groups and both positive controls, in which their presence will cause painful, persistent inflammation, itchiness, and cosmetic defects. Furthermore, successful wound reepithelialization by PE 1.0% on day 11 had revealed the smallest wound scar as compared to the other treatments. This is probably due to actin and myosin (cell bodies of the mature collagen) that were recruited and pulled together from the wound margin until finally reaching and fully occupying the wound bed [17]. Moreover, at day 14 after wounding, wound received with PE 1.0% was found to have an early remodelling phase that was characterized by complete wound closure with no scar and also the formation of hair. Meanwhile, as expected, the positive controls used such as sea cucumber treatment (PCG) and commercial acriflavine (PCA) had promoted the wound to heal faster, which manifested with smaller wound, less scar produced, and faster epithelialization period at days 7 and 14 after wounding when compared to the untreated wound (NO group) as shown in Supplementary Figure S2.

**Figure 1:**
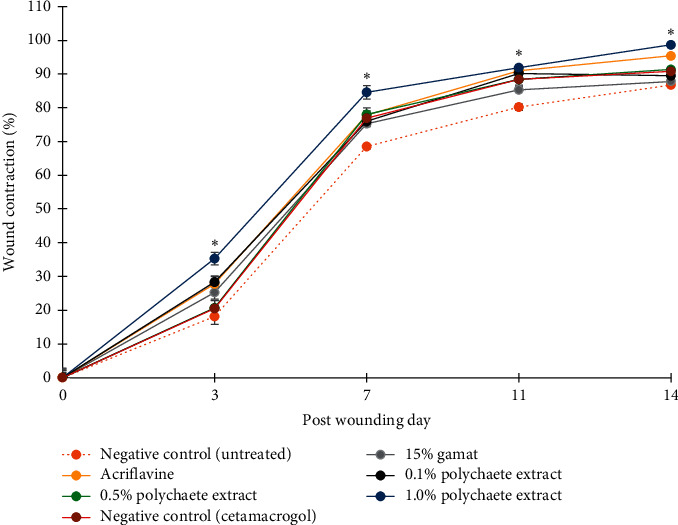
Wound healing percentage (WHP) in different treatment groups was measured on days 3, 7, 11, and 14 after wounding. All treatments were applied once daily on rat's wound for 14 days (NO = no treatment (negative control), PCG = 15% positive control gamat, PCA = 0.1% positive control acriflavine, PE 0.1% = 0.1% polychaete extract, PE 0.5% = 0.5% polychaete extract, PE 1.0% = 1.0% polychaete extract prepared in cetomacrogol ointment, and NC = cetomacrogol ointment (negative control)). The result demonstrated that PE 1.0% showed rapid wound closure as compared with other treatments. Data for WHP from each group is tabulated in Supplementary Table S2. Data was analyzed using one-way ANOVA. ^*∗*^*p* < 0.05 indicates significant difference between PE 1.0% and negative control.

Wound contraction is an essential indicator of wound healing. To measure the wound contraction quantitatively, the value was determined by calculating the wound healing percentage (WHP). Among all, the polychaete extract ointment, PE 1.0%, showed the highest WHP (35.3%) as early on day 3 when compared to other groups (Figure 1). The value has a significant difference when compared to rats from PCG group (25.2%), whereby gamat is a very well-known traditional medicine with wound healing properties. On day 7, the reduction of wound size in all groups reached more than 70% reduction of WHP except for the untreated group (68.4%). The result also suggested rapid wound healing in PE 1.0% (84.6%) compared to other experimental groups. The acute wound contraction was almost complete towards the second week of treatment. Interestingly, wound treated using PE 1.0% again revealed the highest WHP (91.9%) on day 11, which has a significant difference as compared to PCG (85.3%) and NO (80.2%) (Figure 1). However, the WHP in PE 1.0% has no significant difference when compared to the rest of the experimental groups (PE 0.1% and PE 0.5%).

A study by Deng et al. [18] using earthworm (oligochaete), *Lumbricus terrestris*, for wound treatment has also reported a favourable wound healing result. He reported the use of sprayed earthworm's extract (100% v/v) on a 3 mm full thickness wound; twice a day had resulted in 88.34% wound closure on day 11 of treatment. Interestingly, PE 1.0% utilized in our study had demonstrated a better WHP (91.9%) compared to Deng et al. whereby the polychaete ointment was applied only once daily on a larger wound diameter (8 mm) for the same period. Thus, the results suggested that PE 1.0% is more effective for wound healing as compared to earthworm extract. As expected on day 14, PE 1.0% (98.7%) revealed significantly the highest wound contraction compared to NO (86.8%), PE 0.1% (89.5%), PE 0.5% (91.4%), and NC (90.8%). Based on gross macroscopic observation (Supplementary Figure S2) and WHP results (Figure 1), PE 1.0% showed the most effective wound healing ability as compared to other treatments, particularly PCG. In this study, the PE treatment was prepared using only two ingredients: *M. moribidii* extract and cetomacrogol emulsifying ointment. Contrarily, PCG constituent used in this study is composed of other chemical compositions, which are well-known in wound healing activity such as *S. variegatus* (sea cucumber) extract (15% concentration), *Eucalyptus* oil, *Cocos nucifera* oil, and *Myristica fragrans*. Commercial gamat oil was used instead of the sea cucumber crude extract itself, because of limitation in obtaining the live organism for the experiment. Nevertheless, further study using the crude extract of *S. variegatus* should be conducted to compare with *M. moribidii* extract on the acute wound healing effects.

Besides, the highest WHP of PE 1.0% on days 3, 7, 11, and 14 had also probably related to the fatty acids composition that commonly presents in polychaetes, wherein polychaetes' fatty acid comprised of mostly polyunsaturated fatty acids (PUFAs), which serve benefits such as immunoregulatory, anti-inflammatory, and tumour-preventing properties that can help the wound healing process. A study by Alava et al. [19] on the cultured mud polychaete, *M. mossambica* (Eunicidae), revealed that the species has between 7 to 12% PUFA content, consisting of arachidonic acid (AA) (0.2 to 0.5%), eicosapentaenoic acid (EPA) (0.2 to 0.3%), and docosahexaenoic acid (DHA) (0.3 to 0.5%). However, Meunpol et al. [20] reported a higher concentration of AA (7.78%), EPA (7.52%), and DHA (1.34%) in *M. sanguinea*. Nevertheless, a further study needs to be conducted to confirm the presence of PUFA content of *M. moribidii* extract. On the other hand, moist environments could speed up wound healing in an acute wound. As formulated in the PE 1.0%, the ointment was able to trap and retain moisture. This condition had been proven to stimulate the breakdown of dried fibrin and dead tissues, stimulate keratinocyte proliferation, enhance collagen synthesis, and induce angiogenesis [21]. Altogether, these benefits allow a smooth flow of overlapping phases for wound healing in PE 1.0%, hence explained the highest WHP in every treatment period.

### 3.2. Rats Behaviour

Overall, the recorded result suggested that rats in all seven experimental groups (NO, PCG, PCA, PE 0.1%, PE 0.5%, PE 1.0%, and NC) demonstrated various rats' behaviour throughout the experimental period (Table 1). During day 0 to day 3 of this study, all rats were inactive where sleeping and resting activities were prominent in all experimental groups due to pain endured after wound operation. Some rats preferred to stay at the cage corner while some burrow within the cage's bedding with their head or body parts covered. This was due to rat's behaviour to develop empathy feeling, following the other's pain throughout the animal room. The response can be sensed by the individual-caged rat and transferred among the animals [22]. Rats are just like a human, where during pain or wound experience, less appetite was observed, more time was spent for rest and less time allocated for locomotor activities. Hashim et al. supported the current finding where traumatic stress that was induced on rats has led to three days of more resting and sleeping activities [9]. Moreover, sleeping was reported to improve immune response, which helps in the wound healing process by elevating pro-inflammation cytokines activities [23]. As observed in our result, burrowing activity was a typical pain behaviour elicited in rodent after a traumatic surgery, which also indicated a positive sign of recovery. Furthermore, rats' licking and scratching around the wounded area were regularly observed on day 0, soon after recovering from the anaesthetic effect. This is probably due to itchiness and discomfort to SD rats following the wound creation on healthy skin, which happened on SD rats in all groups of treatments. Licking and scratching remove unclean matters, wound contaminants, and infected tissue surrounding the wound area. This behaviour primarily involved saliva, containing vital enzyme, protein, and harmless bacteria which indirectly helps in accelerating the healing process.

Interestingly, on day 7, SD rats in most groups except for NO group started to recover from their inactive state. This time, sleeping and resting activity have ceased and been dominating by cage exploration, especially walking and exploring around the cage. Their wound underwent controls. Active SD rats' movement on day 7 probably concerns the ongoing process in the proliferation phase, which results in smaller wound closure. Therefore, the healing wound perhaps helps to reduce pain and allows for the cage exploration activities. However, the scratching and licking behaviour was prolonged in both groups of negative, indicating insufficient recovery from the wound pain. Nevertheless, Abbasian et al. suggested that the saliva contains many bioactive compounds and licking behaviour in the animal was among the vital activities that will enhance wound healing [24].

On day 7 onwards, all of the tested polychaete ointments (PE 1.0%, PE 0.5%, and PE 1.0%) exhibited similar results as both positive controls (PCG and PCA) through active response and notable cage exploration. This included climbing as seen on days 11 and 14, where climbing was considered as an exercise for rodents that would accelerate anti-inflammatory response, as well as promoting cutaneous wound healing. This suggested a positive healing progression and rats had successfully recovered from the pain injury.

### 3.3. Histological Analysis

**Figure 2:**
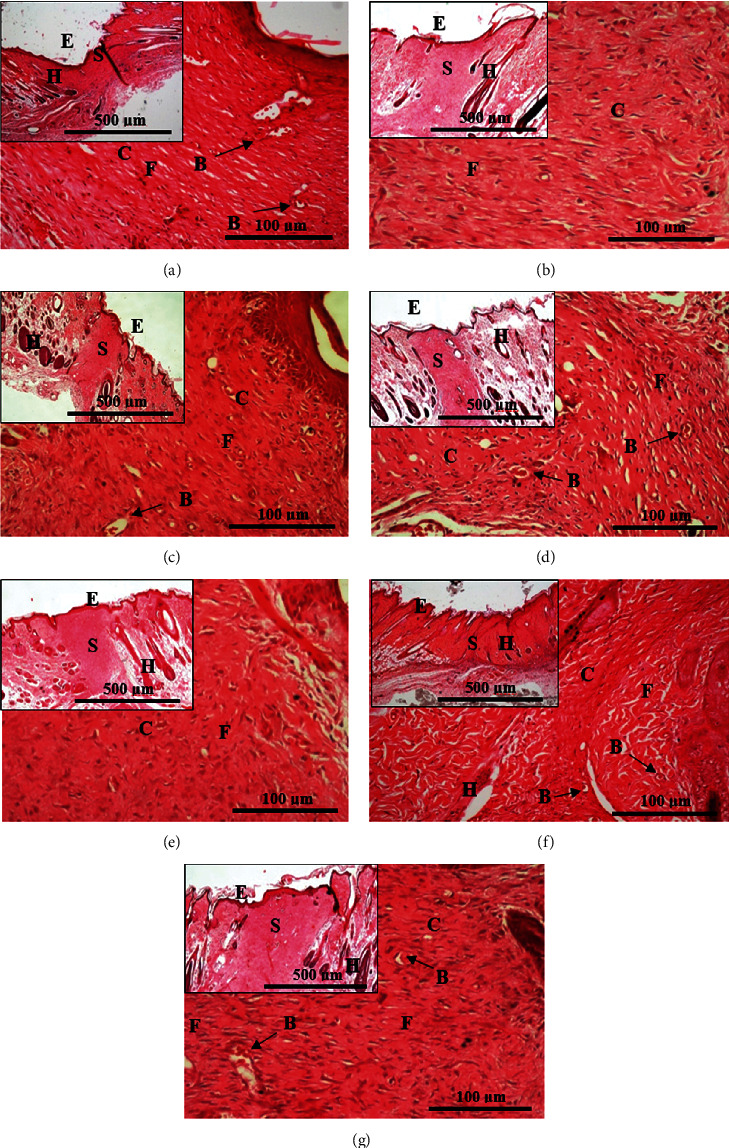
Hematoxylin and eosin stained histopathological section of granulation healing tissue of different groups at day 14. (a) NO = no treatment (negative control), (b) PCG = 15% gamat (positive control), (c) PCA = 0.1% acriflavine (positive control), (d) PE 0.1% = 0.1% polychaete extract, (e) PE 0.5% = 0.5% polychaete extract, (f) PE 1.0% = 1.0% polychaete extract prepared in cetomacrogol ointment, and (g) NC = cetomacrogol ointment (negative control). E = epidermis, S = scar/granulation tissue, C = collagen, F = fibroblast, H = hair follicle, and B = blood vessel. Smaller figure at upper left for each group was indicated by zoomed-out histological tissue at magnification 40x; Bar = 500 *μ*m. Larger size figure in each group indicated the zoomed-in histological skin tissue and 200x; Bar = 100 *μ*m.

On day 14 after wounding, the surrounding skin sample with scar part was excised for further examination through H&E and MT staining. Histopathological analysis further confirms the gross observation, where the untreated wound had slow healing process which manifested with cardinal signs of inflammation in the tissues (reddish), high granulation tissue at the wound gap with no growth of skin appendages as shown in Figure 2. Meanwhile, in the wound treated with polychaete extract PE 1.0%, the tissues had more reorganized fibroblast fibres indicating more developed collagen, with no scar formation, and the emergence of hair follicle which suggested the wound is entering the early remodelling phase. This explained the ability of rapid healing by PE 1.0% that had proved the highest WHP on days 3, 7, 11, and 14 (Figure 1) (Supplementary Table S2). When comparing with the cross-section of excised skin in PE 1.0% (Figure 2(f)), it was observed that the epidermal gap was more prominent in both positive and negative control, as well as in a treated group with polychaete ointment, PE 0.1% (Figure 2(d)) and PE 0.5% (Figure 2(e)). Meanwhile, PE 1.0% had revealed a smaller wound margin. Interestingly, reepithelialization was almost complete in PE 1.0%, where only a minimal scar area was observed when compared with other groups. Indirectly, the healing outcome by PE 1.0% would improve the wound aesthetics with no noticeable scar marked.

Moreover, neovascularisation and angiogenesis have played an essential role in supplying the newly formed granulation tissue with sufficient oxygen and nutrients. This was crucial to ensure the cellular metabolisms proceed at the physiological level and prevent tissue hypoxia when oxygen is depleted. In PE 1.0% group, more emergence of blood vessels was observed in tiny vessels, which were indicated by the structure of blood cells inside (Figure 2(f)). Our result was in accordance with Deng et al. which reports that the use of earthworm extract has developed a large number of blood capillaries that are crucial in wound healing by enhancing blood circulation and nutrient transport, increasing the permeability of blood capillaries, and promoting granulation tissue formations and tissue repair [18]. The presence of neovascularisation thus marked the positive results of wound healing by PE 1.0%.

Besides, a study utilizing another annelid, *Hirudo orientalis* (leech; Hirudinea), by Derestani et al. also demonstrated much hair growth at the recovered horizontal wound (20 mm) [25]. Despite the excellent benefits of leech in literature, the study also reported that the high nutrition from hair follicles probably induced blood circulation at the wounded area [25]. Hence, this supported our finding in PE 1.0% (Figure 2(f)), where the nutritional impact from the emergence of hair follicles might indirectly assist the healing process which is almost completed with WHP = 98.7% on day 14. This also suggested that PE 1.0% had a significant medicinal effect on wound closure and hair growth. Contrarily, histology observation on NO group (Figure 2(a)) resulted in a large wound gap. This was perhaps explained by incomplete wound healing and the failure of granulation tissues to occupy the wounded area to reach the same height of the intact skin.

**Figure 3:**
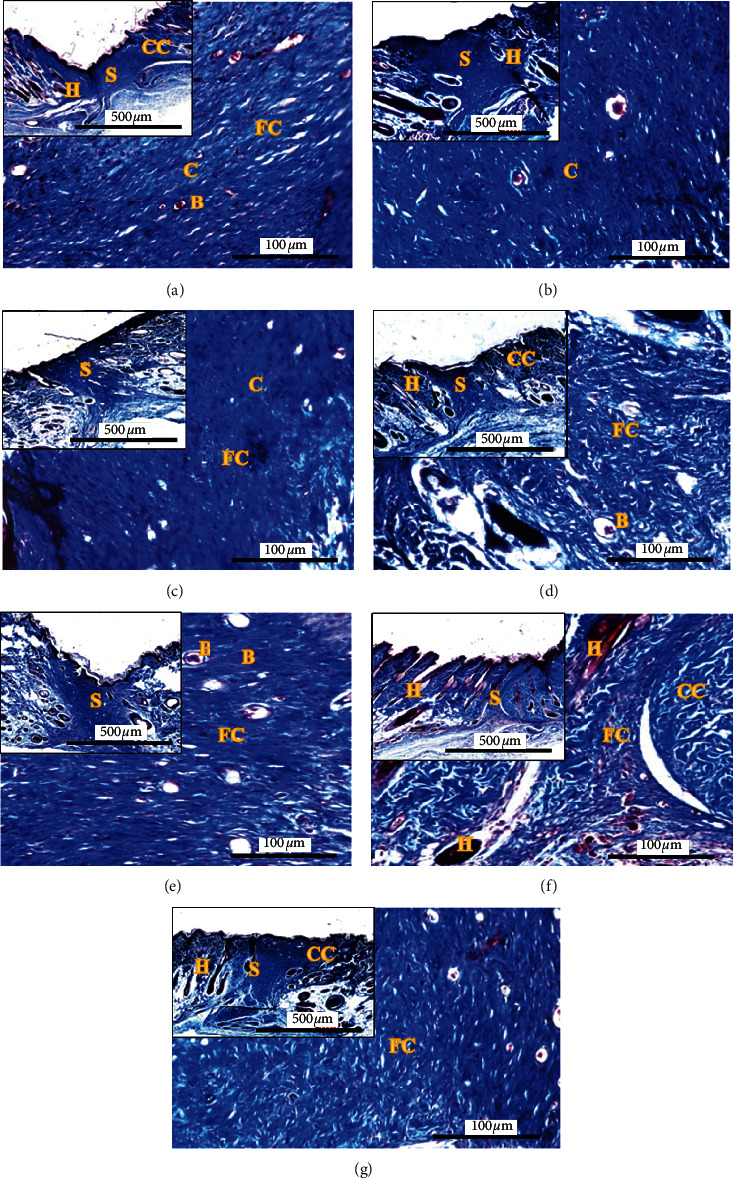
Masson's trichome stained histopathological section of rat's skin tissue of different groups at day 14. (a) No treatment (negative control), (b) PCG = 15% positive control gamat, (c) PCA = 0.1% positive control acriflavine, (d) PE 0.1% = 0.1% polychaete extract, (e) PE 0.5% = 0.5% polychaete extract, (f) PE 1.0% = 1.0% polychaete extract prepared in cetomacrogol ointment, and (g) NC = cetomacrogol ointment (negative control). E = epidermis, S = scar/granulation tissue, C = collagen, F = fibroblast, H = hair follicle, B = blood vessel, CC = coarse collagen, and FC = fine collagen. The smaller figure at upper left for each group was indicated by zoomed-out histological tissue at magnification 40x; Bar = 500 *μ*m. Larger size figure in each group indicated the zoomed-in histological skin tissue and 200x; Bar = 500 *μ*m.

To further confirm the effect of treatment on the collagen deposition and reorganization, MT staining was used. The wound healing process had displayed more apparent collagen arrangement, while collagen density can be differentiated through colour intensities at the wounded area, as shown in Figure 3. Results revealed the parallel arrangement of collagen with the epidermis were visible in both groups: NO (Figure 3(a)) and PE 0.5% (Figure 3(e)). This is an indicator of scar tissue, which is still fragile and easy to retract [26]. In detail, the wound tissues are not fully mature; hence it needs longer healing time to recover and gain tissue strength. Moreover, the histological data was parallel to the wound contraction results (Figures 1 and 2), where NO group has indicated the lowest WHP throughout the treatment period.

Wound treated with PE 1.0% displayed fine collagen arrangement in crosslinking and intertwined manner (Figure 3(f)). This collagen orientation helped in regaining the wound's tensile strength [27]. Moreover, the intense colour of blue stain absorbed with the growth of hair follicles in the treated wound suggested dense fibroblasts and collagen production [11]. To date, about 28 types of collagen had been identified and majorly belong to fibril-forming subfamily. These include type I (80 to 85%) and type III (8 to 11%) collagen within the skin. Type III collagen predominates typically in an early progression during the proliferation phase, but as it matures, it is replaced with stronger type I collagen [28]. Apart from that, PE 1.0% collagens' fibril was observed to have a closed structure with the unwounded dermis (CC = coarse collagen), which were characterized in a basket weave pattern. This marked the outcome of functional connective tissue matrix after the treatment [26]. Thus, our finding in PE 1.0% group indicated that the type III collagen had been replaced with stronger and mature type I collagen. However, both positive controls, PCG (Figure 3(b)) and PCA (Figure 3(c)), failed to display a better collagen orientation and arrangement, in addition to the emergence of skin appendages as found in PE 1.0% group. In other words, rats in PE 1.0% treated groups showed rapid wound healing with mature collagen that successfully filled the ECM compared to other groups.

### 3.4. Safety Assessment of *Marphysa moribidii* Ointment

Skin irritancy, microbial contamination, and heavy metal detection test were included to assess the safety quality of *M. moribidii* aqueous extract emulsifying ointment for cutaneous use. Skin irritancy is a compulsory test to provide scientific proof regarding the safety of a new product or formulation before it can be marketed. Until the present date, no study reported the safety or toxicity effects of *M. moribidii* as a wound treatment. The present results suggested that *M. moribidii* ointment has no irritation effect on the skin, which was indicated by “0” score for oedema and erythema (Supplementary Table S3). Oedema is the swelling of tissue formed by the body's fluids, while erythema is caused by hyperemia—excess blood flow in the blood capillaries that can be observed by redness on the skin. These are the noticeable symptoms that can be used as an early diagnosis on the harmful effects of the ointment. The “0” score in all polychaete concentrations was probably due to the absence of chemical used along with the manufacture of the *M. moribidii* ointment.

In this study, deionized water was the only solvent used for polychaete extraction. Plus, water has been used as a favourable solvent in extracting traditional medicine. This is because water is considered safe and cheap, thus being able to extract targeted compounds, in addition to its greener and environment-friendly effects. Therefore, the use of water as the universal solvent was expected to reduce any side effects or irritation potential. Meanwhile, similar 0 scores were recorded in the NC group, hence confirming that the petroleum-based ointment is safe as a vehicle for the treatment delivery. Moreover, it also helps to absorb and remain on the skin at a longer time, retain moisture, and minimize skin dryness by securing the oily layer that traps the available water beneath, thus preventing water loss from the skin surface.

Ensuring product safety for consumers' use is high priority before it can be commercialized. Thus, MCT was also done to check the level of contamination caused by bacteria and fungi, as well as the detection of specific pathogenic microbes that may grow on the tested sample. The PE 1.0%, which had been found the most effective concentration in the previous section (Figure 1 and Supplementary Figure S2), was subjected to MCT. According to the guideline from British Pharmacopoeia (BP), the accepted threshold value for a nonsterile dosage sample is lower than 10^2^ CFU/g and 10^1^ CFU/g for TAMC and TYMC value, respectively, with the absence of growth of *P. aeruginosa* and *S. aureus*. Interestingly, both TAMC and TYMC values from the PE were not detected (ND) (Supplementary Table S1). Moreover, the sample also did not support the growth of *P. aeruginosa* and *S. aureus*, which are the most common isolated bacteria from a wounded skin [7]. Furthermore, the ointment would hinder severe illness to the patient by preventing wound contamination as well as the potential bacterial colonization (especially *P. aeruginosa* and *S. aureus*) on the patient's wound. The MCT results demonstrated that PE 1.0% had successfully passed all the criteria set by the BP guidelines and hence suggested the potential of aqueous extract emulsifying ointment of *M. moribidii* in wound healing for topical cutaneous application.

Apart from that, the results for heavy metal detection also demonstrated no detection of As, Cd, Pb, and Hg in the PE ointment (Supplementary Table S4). Studies had found that these naturally occurred metals can be fatal to human health if present at high concentrations. These toxic elements are commonly found in cosmetic and personal care products. The elements can absorb and enter the bloodstream via the skin barrier. Prolonged use will cause heavy metal accumulation in the body and may lead to a number of undesirable complications. For instance, Pb can have adverse effects on the neuron system and cause the behaviour, language, and learning disabilities [29]. Also, Hg had been associated with nervous and immune system toxicity and reproductive as well as respiratory toxicity. Cd accumulation can lead to kidney failure, reproductive disorder, and lungs disease and may affect the bones [30]. Meanwhile, As can result in nephrotoxicity, hepatotoxicity, and neurotoxicity at high exposure on vital organs. Hence, this study had successfully demonstrated the safe usage of PE 1.0% on the cutaneous wound with no health-threatening issues. Moreover, the results for all heavy metals are lower than the permissible limit propounded by the National Pharmaceutical Regulatory Division (NPRA), which can ensure more safety levels to the consumers. Altogether, *M. moribidii* extract emulsifying ointment can be regarded as safe for prolonged use with no hazardous effects when applied on wounded skin.

### 3.5. Antimicrobial Evaluation

Marine organisms commonly produce a wide array of biologically active compounds as well as developing antimicrobial activity to ensure survival against the harsh environment. However, the presence of antimicrobial activity in wound medication had allowed for better wound cleansing by inhibiting bacterial colonization. Moreover, bacterial proliferation and imbalance would affect the subsequent process causing failure in wound healing [31]. Hence, the presence of antibacterial activity would be an added value to the rapid healing demonstrated by PE 1.0%. However, the current finding showed that *M. moribidii* aqueous extract exhibited weak antibacterial activity against the pathogenic bacteria such as *S. aureus, S. epidermidis, P. aeruginosa*, and *K. pneumonia* (Table 2). For example, the MIC value of the PE on *E. coli* was 0.4 g/ml, whereas the MBC value was more than 0.4 g/ml (Table 2). An attempt to increase the polychaete extract into a higher concentration (1.0 g/ml polychaete stock solution) was likely unattainable due to solubility problems. Consequently, the status of the *M. moribidii* extract as bactericidal or bacteriostatic remains unknown due to inadequate information to determine the accurate MBC to MIC ratio.

Besides these limitations, PE 1.0% had proved an outstanding result of WHP, with almost 100% healing on the 14^th^ day of treatment (Figure 1) (Supplementary Table S2). The underlying cause of weak antibacterial activity in PE 1.0% was probably due to the selection of water as a solvent for the extraction procedure. The water seemed ineffective in extracting more antibacterial compounds from *M. moribidii*, where 0.4 g/ml had shown insufficient value to inhibit bacterial growth. Nevertheless, the solubility problem during the polychaete extraction might be overcome in the future by choosing other solvents such as methanol and ethanol [31, 32]. These solvents probably have higher abilities to extract more bioactive compounds from the polychaete extract with antibacterial properties. Previous studies showed that the use of organic solvents resulted in positive results in the antibacterial test. For instance, ethanol extract of other polychaete species, *Halla parthenopeia*, *Hydroides elegans*, and *Perinereis cultifera* showed positive antibacterial effect against *P. aeruginosa*, *Bacillus cereus*, and *E. coli*, respectively [32, 33]. *Arenicola marina*, a coastal polychaete, was the first finding reported to produce arenicin-1, an antimicrobial peptide, which assembles on the negatively charged bacterial membrane and gives a lethal effect on the microbes [32]. Thus, the ability of aqueous *M. moribidii* extract to combat pathogenic bacteria is required to be confirmed through other studies.

### 3.6. Detection of Chemical and Metabolites in Aqueous *Marphysa moribidii* Extract

Chemical screening such as phytochemistry is a qualitative test, which is generally conducted on the plant origins for detection of secondary metabolites. This includes the test for alkaloids, flavonoids, terpenoids, and saponins. Thus, the screening process was conducted using *M. moribidii* aqueous extract due to its close contact with the mangrove nature. Hence, the current study intended to discover the possible phytochemical and metabolite compounds presence from *M. moribidii* that would be beneficial to health, especially in wound healing.

Chemical tests and ^1^H NMR analysis have revealed various bioactive compounds that were detected in *M. moribidii* extract that possesses medicinal effects in treating wound injuries. Through the chemical tests, *M. moribidii* aqueous extract revealed the presence of some classes of biologically active compounds such as alkaloid, flavonoid, saponin, and terpenoid (Supplementary Figure S3). The presence of bioactive compounds such as alkaloids and terpenoids in *M. moribidii* aqueous extract might explain its pharmacological properties that can assist in the wound healing process, including showing anti-inflammatory, antimicrobial, antioxidant, and radical scavenging activities [34]. During wound healing, the bioactive compounds accelerate antioxidant concentration in the wound, strengthen the skin, and reduce inflamed tissue, hence inducing blood flow. For example, flavonoid is known for its antioxidant properties due to the presence of phenolic groups in its molecular structure. Meanwhile, saponin is responsible for elevating the epithelialization rate during wound closure by its antimicrobial and antioxidant activities [35].

During acute wound injuries, reactive oxygen species (ROS) was developed at the wound site as host defence. The failure of sufficient antioxidant production to detoxify and balance the average ROS level in the acute wound (100 to 250 *μ*M) could lead to a detrimental effect on a nonhealing injury [36]. Hence, antioxidant property from flavonoid and saponin in the polychaete extract probably supports its effective healing results. Gamat oil utilized in this study from species *S. hermanni* also contains flavonoid, saponin, and terpenoid compounds. In addition to other bioactive compounds, the healing process of gamat oil was assisted by its high sulfated glycosaminoglycan content that resides in the integument body wall of gamat [37]. However, based on histological analysis, the treatment showed to be less effective when compared to PE, whereas natural compounds such as alkaloid, terpenoids, flavonoid, and saponin were absent from synthetic wound medicine like acriflavine. Thus, the medicine probably lacks antioxidant property or other inherent abilities that would enhance cells' proliferation and epithelialization in the wound. The primary function of flavine in synthetic wound medicine is to clean the wound from pathogenic bacteria. However, its ability is not sufficient to demonstrate better results compared to PE. Furthermore, *M. moribidii* mainly inhabits the upper tidal flats area of Morib area, around and inside the hard mangrove slits roots of *Rhizophora apiculata*. Plus, *M. moribidii* exhibited a subsurface deposit feeder, which consumes a high amount of organic matter left on the mangrove land. This probably is composed of dead leaves, plants, and other organisms which had shown positive results in this study. Amazingly, intestinal contents of this polychaete also revealed a high percentage of organic matter consumed [5]. Hence, the secondary metabolites detected in this study probably were contributed from the ingestion of plant-based materials as its daily diet. This finally nourished and make up the polychaetes' body with useful metabolites.

Details of the bioactive compound available in the polychaete extract were identified using ^1^H NMR analysis. The analysis is a robust technique that can be used to identify bioactive compounds in the mixture of the sample. In this study, the ^1^H NMR spectrum (Supplementary Figure S4) of *M. moribidii* extract showed the presence of compounds from various classes including amino acids, organic acids, nitrogenous compounds, vitamin, purine derivatives, and fatty acids. The amino acids identified according to its ^1^H NMR signals were phenylalanine, betaine, glycine, methionine, taurine, and guanidinoacetate. Phenylalanine was identified based on the signals observed at *δ*_H_ 7.34 (m), 7.30 (m), and 7.26 (m). Singlets at *δ*_H_ 3.56 and 3.79 were tentatively assigned as glycine and guanidinoacetate, respectively. The characteristic signals for taurine were also observed at *δ*_H_ 3.14 (t) and 2.89 (t) along with betaine at *δ*_H_ 3.29 (s) and 3.89 (s). Multiplets signals for methionine were also identified at *δ*_H_ 3.89 (m), 2.69 (m), 2.2 (m), and 2.04 (m). The ^1^H NMR spectrum showed the signals for organic acids including succinate at *δ*_H_ 2.36 (s) and lactate at *δ*_H_ 4.33 (m) and 1.34 (d) as well as acetate *δ*_H_ 1.92 (s). Some nitrogenous compounds identified were creatinine at *δ*_H_ 3.02 (s) and 4.05 (s), trimethylamine at *δ*_H_ 2.99 (s), dimethylamine at *δ*_H_ 2.75 (s), and trimethylamine at N-oxide *δ*_H_ 3.45 (s).

Some derivatives of bromophenols were also identified in *M. moribidii.* The symmetrical protons of the benzene ring of 4-bromophenol were represented by two doublet peaks located at *δ*_H_ 7.11 and 6.79. The compound 2,4,6-tribromophenol was also identified based on the characteristic signals at *δ*_H_ 7.77 (s) and 7.72 (s). Apart from this, in the ^1^H NMR spectrum was also observed the typical signals of fatty acids, including the signals at *δ*_H_ 2.36 (s) and 1.28 (s) attributed to 3-hydroxyisobutyrate as well as at *δ*_H_ 2.29 (m), 1.21 (d), 4.15 (m), and 2.30 (m) of the 3-hydroxybutyrate. Details of the metabolites assignment are summarized in Table 3. The identified compounds, such as glycine and methionine, which are the simplest amino acid, were responsible for wound healing properties. These compounds function in the building block of protein for collagen synthesis. Meanwhile, taurine, with its antiapoptotic, anti-inflammatory, and antioxidant activity, is also useful in rapid cell epithelialization [38]. Apart from that, a study conducted by Sá et al. reported that glycine-supplemented hamsters revealed high production of type I instead of type III collagen [39].

In a normal collagen maturation, fibroblasts initially synthesize collagen type III, followed by the replacement by type I collagen, which is more mature and thicker; thus, mature type I collagen that was visible in MT staining was probably synthesized due to the presence of glycine content in the polychaete extract, which helps in mature skin regeneration. In general, most wounds develop moderate tissue hypoxia, a condition of oxygen-deficient reaching to tissue cells due to damage or lack of blood vascularisation on the wound area. Hypoxic wound naturally generates high lactate production through aerobic glycolysis, especially during the inflammation phase of healing. Then, lactate accumulation helps in initiating angiogenesis and enhances collagen synthesis and migration. Hence, lactate content in *M. moribidii* extract would add more benefits in generating angiogenesis and collagen deposition, as seen in histological tissue slide of PE 1.0% (Figure 3(f)). Besides, organic acids like acetate and fatty acids, including 3-hydroxybutyrate and 3-hydroxyisobutyrate, were also detected in *M. moribidii* aqueous extract.

The halogenated aromatics are classified as toxic substances involved in the defensive mechanism or as a result of interaction between microorganisms. Hence, 4-bromophenol and 2,4,6-tribromophenol probably synthesized by *M. moribidii* to increase survivability from injuries caused by predators in its habitat. Bromophenol is a typical marine metabolite, and interestingly, the compound also had been reported by Coutinho et al. in polychaetes such as *M. macintoshi and M. sanguinea* with antibacterial benefits [40]. However, the effect cannot be observed in our antibacterial tests, perhaps due to low bromophenol constituent in the *M. moribidii* extract. Other metabolites that were detected include hypoxanthine and trigonelline. Hypoxanthine is the intermediate in the metabolism of adenosine that can be found in all living species, ranging from bacteria to humans. Trigonelline is known as a product of the metabolism of niacin (vitamin B3) which could also contribute to the bioactivity in *M. moribidii*. Through some of the stated compounds and their function in wound healing, it is inarguable more that polychaete is a unique creature that comprises useful metabolites from primary and secondary classes. The ability of enhanced wound closure in all SD rats treated with the polychaete extract without any side effects has confirmed the positive effects of *M. moribidii* to be utilized as a choice for wound treatment.

## 4. Conclusions

This study has provided and established new knowledge regarding the utilization of *M. moribidii* as a potential wound healing agent. Despite low antibacterial activity shown against bacteria, the PE 1.0% has revealed almost complete wound closure, rapid wound healing, mature collagen deposition, and minimal scar detection. Plus, the application of polychaete ointment has restored the normal behaviour with no adverse effect on the rats' well-being. Moreover, the use of water as an organic solvent, absence of skin irritation, and bacterial colonization, as well as no heavy metals constituent have ensured the polychaete formulations as safe to be applied by consumers. The participation of beneficial compounds from various classes, including alkaloid, terpenoid, flavonoid, saponin, amino acids, organic acids, nitrogenous compounds, and others, have probably functioned synergistically, which assisted in the wound healing process. In summary, the use of PE 1.0% would provide an alternative solution for rapid wound healing, thus overcoming the acute wound problems that might turn into a chronic nonhealing wound.

## Figures and Tables

**Table 1:** Rat's behaviour in an individual cage for 30 min of observation in the morning throughout 14 days of wound healing using several treatments.

Treatment	Activity/day	0	3	7	11	14
NO	Wound licking/scratching	+	+	+	−	−
	Sleeping/resting	+	+	+	−	−
	Cage exploration (walking/climbing)	−	−	+	+	+

PCG	Wound licking/scratching	+	+	−	−	−
	Sleeping/resting	+	+	−	−	−
	Cage exploration (walking/climbing)	−	−	+	+	+

PCA	Wound licking/scratching	+	+	−	−	−
	Sleeping/resting	+	+	−	−	−
	Cage exploration (walking/climbing)	−	−	+	+	+

PE 0.1%	Wound licking/scratching	+	+	−	−	−
	Sleeping/resting	+	+	−	−	−
	Cage exploration (walking/climbing)	−	−	+	+	+

PE 0.5%	Wound licking/scratching	+	+	−	−	−
	Sleeping/resting	+	+	−	−	−
	Cage exploration (walking/climbing)	−	−	+	+	+

PE 1.0%	Wound licking/scratching	+	+	−	−	−
	Sleeping/resting	+	+	−	−	−
	Cage exploration (walking/climbing)	−	−	+	+	+

NC	Wound licking/scratching	+	+	+	−	−
	Sleeping/resting	+	+	−	−	−
	Cage exploration (walking/climbing)	−	−	+	+	+

Rats' wounds were treated with NO = no treatment (negative control), PCG = 15% positive control gamat, PCA = 0.1% positive control acriflavine, PE 0.1% = 0.1% polychaete extract, PE 0.5% = 0.5% polychaete extract, PE 1.0% = 1.0% polychaete extract prepared in cetomacrogol ointment, and NC = cetomacrogol ointment (negative control). *n* = 5. − = no activity; + = presence of activity.

**Table 2:** The value of MIC, MBC, and MIC to MBC ratio of *Marphysa moribidii* aqueous extract against five selected bacteria. MIC value for *Escherichia coli* was 0.4 g/ml, but probably higher than 0.4 g/ml for *Staphylococcus aureus*, *S. epidermidis*, *Pseudomonas aeruginosa*, *and Klebsiella pneumonia*.

Microorganisms	Aqueous extract of *Marphysa moribidii*			Remark
	MIC (g/ml)	MBC (g/ml)	MBC: MIC	
*S. aureus*	>0.4	>0.4	ND	*∗*Bactericidal/bacteriostatic
*S. epidermidis*	>0.4	>0.4	ND	*∗*Bactericidal/bacteriostatic
*E. coli*	0.4	>0.4	>1	*∗*Bactericidal/bacteriostatic
*P. aeruginosa*	>0.4	>0.4	ND	*∗*Bactericidal/bacteriostatic
*K. pneumonia*	>0.4	>0.4	ND	*∗*Bactericidal/bacteriostatic

ND = not detected. *∗*Bactericidal/bacteriostatic = the status of *M. moribidii* aqueous extract being bactericidal or bacteriostatic was unable to be determined due to inadequate information.

**Table 3:** Tentative metabolites identification in *Marphysa moribidii* aqueous extract using ^1^H NMR analysis showed various metabolites from various groups, such as amino acids, organic acids, nitrogenous compounds, vitamin, purine derivative, and fatty acids.

Group	Metabolite	Chemical shift (ppm)
Amino acids	Phenylalanine	7.34 (m), 7.30 (m), 7.26 (m)
	Betaine	3.29 (s), 3.89 (s)
	Glycine	3.56 (s)
	Methionine	3.89 (m), 2.69 (m), 2.2 (m), 2.04 (m)
	Taurine	3.14 (t), 2.89 (t)
	Guanidinoacetate	3.79 (s)

Organic acids	Succinate	2.36 (s)
	Lactate	4.33 (m), 1.34 (d)
	Acetate	1.92 (s)

Nitrogenous compounds	Creatinine	3.02 (s), 4.05 (s)
	Trimethylamine	2.99 (s)
	Dimethylamine	2.75 (s)
	Trimethylamine N-oxide	3.45 (s)

Halogenated aromatics	4-Bromophenol	7.11 (d), 6.79 (d)
	2,4,6-Tribromophenol	7.77 (s), 7.72 (s)

Vitamin	Trigonelline	9.21 (s), 8.96 (m), 8.11 (m), 4.42 (s)

Purine derivative	Hypoxanthine	8.16 (s), 8.05 (s)

Fatty acids	3-Hydroxyisobutyrate	2.36 (s), 1.28 (s)
	3-Hydroxybutyrate	2.29 (m), 1.21 (d), 4.15 (m), 2.30 (m)

s = singlet, d = doublet, t = triplet, and m = multiple.

## Data Availability

The data used to support the findings in this study are included in the supplementary materials.

## Supplementary Materials

Supplementary Materials Figure S1: evaluation of antibacterial activity for *M. moribidii* extract against bacterial strains such as *S. aureus*, *S. epidermidis*, *E. coli*, *P. aeruginosa*, and *K. pneumonia* was done using the broth microdilution assay (MIC). The starting aqueous *M. moribidii* extract concentration is at 0.4 g/ml. Three bacterial species were tested (*S. aureus*, columns 1 to 3; *S. epidermidis*, columns 5 to 7; *E. coli*, columns 9 to 12) with concentrations ranging from 0.4 g/ml (row A) to 6.25 mg/ml (row G). About 50 *μ*l of the first bacterial suspension was dispensed into each well (first three columns of the well plate), making up 100 *μ*l as the final well volume and then followed by the other bacterial strains. Row H was utilized as the growth control (sterile MHB) to allow bacterial growth, without the presence of the antibacterial agent. The test for *P. aeruginosa* and *K. pneumonia* was included on another 96-well plate with positive control. Then, the plate was incubated at 37°C for about 18 to 24 h. Figure S2: gross observation of wound contraction on days 0, 3, 7, 11, and 14 following wound creation between different treatment groups. NO = no treatment (negative control), PCG = 15% positive control gamat, PCA = 0.1% positive control acriflavine, and PE 0.1% = 0.1%. Figure S3: chemical test using *M. moribidii* aqueous extract revealed the positive result of the alkaloid, terpenoid, saponin, and flavonoid. These useful metabolite compounds are known for their function in wound healing. Note: “+” sign indicates the presence of compounds. Figure S4: ^1^H NMR spectrum of *Marphysa moribidii* extract. Table S1: the utilization of PE 1.0% for MCT has revealed a positive outcome with the absence of microbes that would probably contaminate the ointment. All results fulfilled the accepted criteria set up by British Pharmacopoeia for microbiological quality for a nonsterile dosage indicating that PE 1.0% ointment is safe for cutaneous uses. Table S2: wound area (mm^2^) and percentage wound contraction for different treatment groups on days 0, 3, 7, 11, and 14 following wound creation between different treatment groups. Table S3: skin irritation score was recorded as “0” according to OECD guideline due to no oedema and erythema formation after rat's skin was applied with different treatments for 1, 24, 48, and 72. Treatments utilized for skin irritation test were NC = cetomacrogol ointment (negative control), PE 0.1% = 0.1% polychaete extract, PE 0.5% = 0.5% polychaete extract, and PE 1.0% = 1.0% polychaete extract prepared in cetomacrogol ointment. Table S4: hazardous materials in aqueous extract *Marphysa moribidii* emulsifying ointment (1.0% w/w). The result showed no detection of heavy metals such as arsenic, lead, cadmium, and mercury in the polychaete ointment.
