# Supplementary material for: Effectiveness of Aqueous Extract of Marine Baitworm Marphysa moribidii Idris, Hutchings and Arshad, 2014 (Annelida, Polychaeta), on Acute Wound Healing Using Sprague Dawley Rats
Source: Evid Based Complement Alternat Med. 2020 Nov 25;2020:1408926. doi: 10.1155/2020/1408926 (PMC7710401; doi:10.1155/2020/1408926)
Supplement: Supplementary Materials — Figure S1: evaluation of antibacterial activity for M. moribidii extract against bacterial strains such as S. aureus, S. epidermidis, E. coli, P. aeruginosa, and K. pneumonia was done using the broth microdilution assay (MIC). The starting aqueous M. moribidii extract concentration is at 0.4 g/ml. Three bacterial species were tested (S. aureus, columns 1 to 3; S. epidermidis, columns 5 to 7; E. coli, columns 9 to 12) with concentrations ranging from 0.4 g/ml (row A) to 6.25 mg/ml (row G). About 50 μl of the first bacterial suspension was dispensed into each well (first three columns of the well plate), making up 100 μl as the final well volume and then followed by the other bacterial strains. Row H was utilized as the growth control (sterile MHB) to allow bacterial growth, without the presence of the antibacterial agent. The test for P. aeruginosa and K. pneumonia was included on another 96-well plate with positive control. Then, the plate was incubated at 37°C for about 18 to 24 h. Figure S2: gross observation of wound contraction on days 0, 3, 7, 11, and 14 following wound creation between different treatment groups. NO = no treatment (negative control), PCG = 15% positive control gamat, PCA = 0.1% positive control acriflavine, and PE 0.1% = 0.1%. Figure S3: chemical test using M. moribidii aqueous extract revealed the positive result of the alkaloid, terpenoid, saponin, and flavonoid. These useful metabolite compounds are known for their function in wound healing. Note: “+” sign indicates the presence of compounds. Figure S4: 1H NMR spectrum of Marphysa moribidii extract. Table S1: the utilization of PE 1.0% for MCT has revealed a positive outcome with the absence of microbes that would probably contaminate the ointment. All results fulfilled the accepted criteria set up by British Pharmacopoeia for microbiological quality for a nonsterile dosage indicating that PE 1.0% ointment is safe for cutaneous uses. Table S2: wound area (mm2) and percentage wound contracti [file 1408926.f1.docx]

**Supplementary materials**

**Effectiveness of Aqueous Extract of Marine Baitworm *Marphysa moribidii* Idris, Hutchings and Arshad, 2014 (Annelida, Polychaeta) on Acute Wound Healing Using Sprague Dawley Rats**

Hannah Syahirah Rapi,^1^ Nor ‘Awatif Che Soh,^1^ Nurul Shahirah Mohd Azam,^1^ M Maulidiani,^1^ Suvik Assaw,^2^ Mohd Nizam Haron,^3^ Abdul Manaf Ali,^4^ Izwandy Idris,^5^ and Wan Iryani Wan Ismail^1^

^1^ Cell Signaling and Biotechnology Research Group (CeSBTech), Faculty of Science and Marine

Environment, Universiti Malaysia Terengganu, 21030 Kuala Nerus, Terengganu, Malaysia

^2^ Faculty of Science and Marine Environment, Universiti Malaysia Terengganu, 21030 Kuala

Nerus, Terengganu

^3^ School of Animal Science, Faculty of Bioresources and Food Industry, Universiti Sultan

Zainal Abidin, 22200, Besut, Terengganu, Malaysia

^4^ School of Agriculture Science and Biotechnology, Faculty of Bioresources and Food Industry,

Universiti Sultan Zainal Abidin, 22200 Besut, Terengganu, Malaysia

^5^ South China Sea Repository and Reference Centre, Institute of Oceanography and

Environment (INOS), Universiti Malaysia Terengganu, 21030, Kuala Nerus Terengganu,

Malaysia

Correspondence should be addressed to Wan Iryani Wan Ismail; [waniryani@umt.edu.my](mailto:waniryani@umt.edu.my)


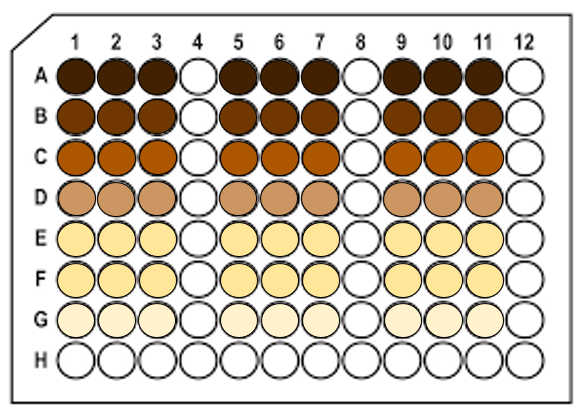


FIGURE S1: Evaluation of antibacterial activity for *M. moribidii* extract against bacterial strain such as *S. aureus, S. epidermidis*, *E. coli, P. aeruginosa* and *K. pnemoniae* were done using the broth microdilution assay (MIC). The starting aqueous *M. moribidii* extract concentration is at 0.4 g/ml. Three bacterial species were tested (*S. aureus*, columns 1 to 3; *S. epidermidis*, columns 5 to 7; *E. coli*, columns 9 to 12) with concentrations ranging from 0.4 g/ml (row A) to 6.25 mg/ml (row G). About 50 µl of the first bacterial suspension was dispensed into each well (first three columns of the well plate), making up 100 µl as the final well volume then followed by the other bacterial strains. Row H was utilised as the growth control (sterile MHB) to allow bacterial growth, without the presence of the antibacterial agent*.* The test for *P. aeruginosa* and *K. pnemoniae* was included on another 96-well plate with positive control. Then, the plate was incubated at 37 ⁰C for about 18 to 24 h.


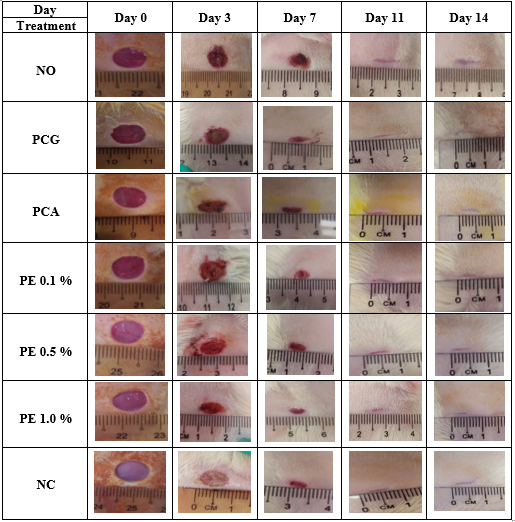


FIGURE S2: Gross observation of wound contraction on day 0, 3, 7, 11 and 14 following wound creation between different treatment groups. NO = no treatment (negative control), PCG = 15% positive control gamat, PCA = 0.1% positive control acriflavine, PE 0.1% = 0.1% polychaete extract, PE 0.5% = 0.5% polychaete extract, PE 1.0% = 1.0% polychaete extract prepared in cetomacrogol ointment, and NC = cetomacrogol ointment (negative control).

**
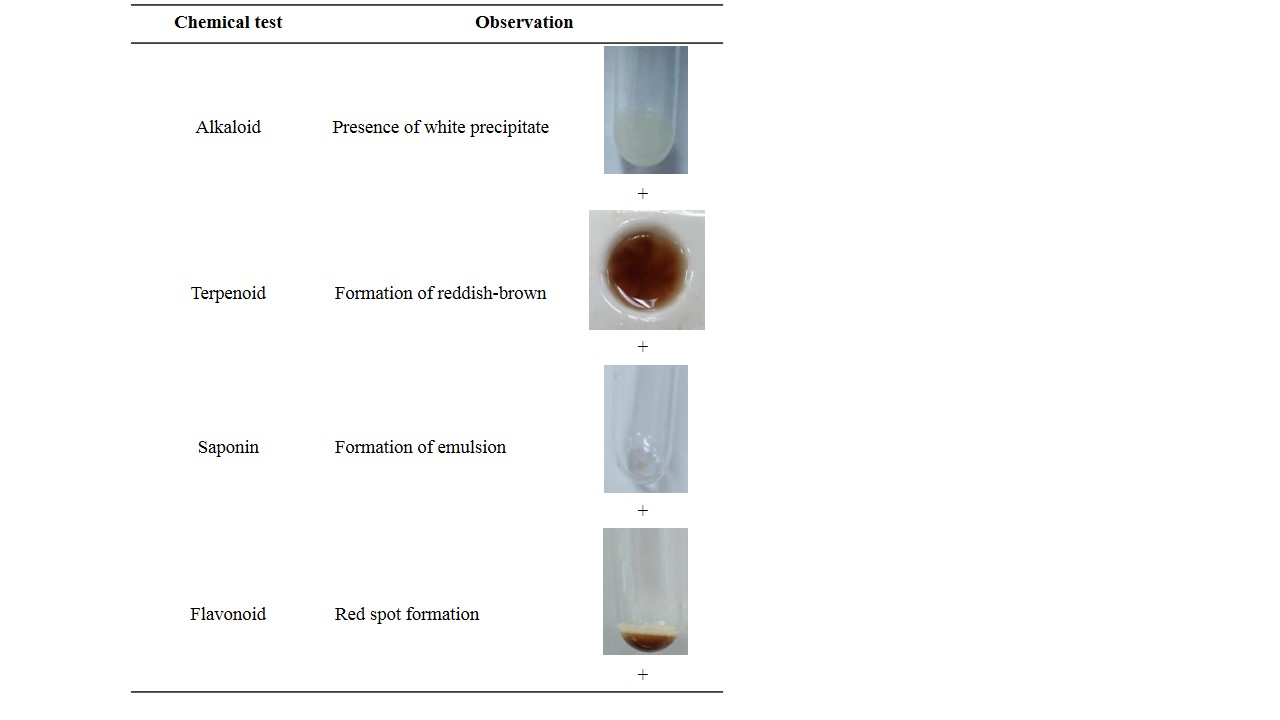
**

FIGURE S3: Chemical test using *M. moribidii* aqueous extract revealed the positive result of the alkaloid, terpenoid, saponin and flavonoid. These useful metabolite compounds are known for its function in wound healing. Note: ‘+’ sign indicates the presence of compounds.

**
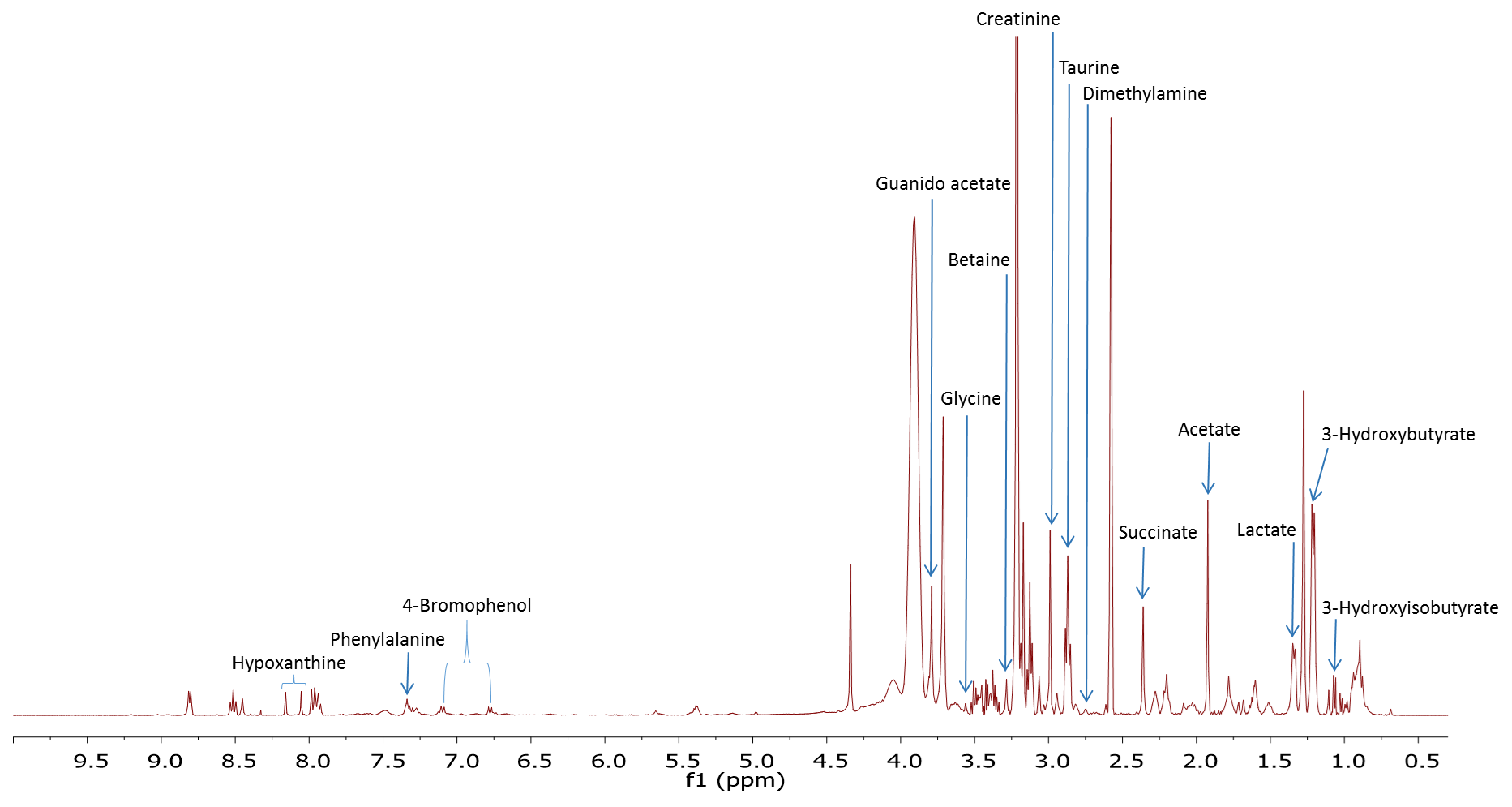
**

FIGURE S3: ^1^H NMR spectrum of *Marphysa moribidii* extract

TABLE S1: The utilisation of PE 1.0% for MCT has revealed a positive outcome with the absence of microbes that would probably contaminate the ointment. All results fulfilled the accepted criteria set up by British Pharmacopoeia for microbiological quality for a non-sterile dosage indicating PE 1.0% ointment is safe for cutaneous used.

| **Test description** | **Unit** | **Result** | **Acceptance criteria for cutaneous used** |
| --- | --- | --- | --- |
| Total Aerobic Microbial Count (TAMC) | cfu/g | ND | 10^2^ |
| Total Yeast & Mold Count (TYMC) | cfu/g | ND | 10^1^ |
| *Pseudomonas aeruginosa* |  | Absent in 0.1 g | Absent in 1 g |
| *Staphylococcus aureus* |  | Absent in 0.1 g | Absent in 1 g |

Note: ND = Not detected.

TABLE S2: Wound area (mm^2^) and percentage wound contraction for different treatment groups on day 0, 3, 7, 11 and 14 following wound creation between different treatment groups.

| **Post wounding days** | **Treatments** | | | | | | | |
| --- | --- | --- | --- | --- | --- | --- | --- | --- |
|  | **Wound area (mm^2^) and wound contraction (%)** | | | | | | | |
|  | **NO** | **PCG** | **PCA** | **PE 0.1%** | **PE 0.5%** | **PE 1.0%** | **NC** | **p value** |
| **Day 3** | 52.3 ± 4.08  (18.2%)^a^ | 47.9 ± 2.3  (25.2%)^a,b^ | 46.2± 3.3  (27.9%)^b,c^ | 45.8± 3.8  (28.4%)^b,c^ | 50.8 ± 1.4  (20.7%) ^a,b^ | 41.4 ± 1.8  (35.3%)^c^ | 50.9 ± 3.1  (20.5%)^a,b^ | 0.000 |
| **Day 7** | 20.2 ± 3.4  (68.4%) ^a^ | 15.9 ± 2.8  (75.3%)^a,b^ | 14.2 ± 3.3  (77.9%)^a,b^ | 15.4 ± 2.2  (75.9%) ^a,b^ | 14.1 ± 3.1  (77.9%)^b^ | 9.9 ± 2.6  (84.6%)^b^ | 14.8 ± 3.5  (76.9%)^a,b^ | 0.001 |
| **Day 11** | 12.7 ± 0.8  (80.2%) ^a^ | 9.5 ± 1.3  (85.3%)^a,b^ | 5.8 ± 1.7  (90.9%)^b,c^ | 6.3 ± 1.9  (90.2%)^b,c^ | 7.4 ± 3.0  (88.4%)^b,c^ | 5.2 ± 2.9  (91.9%)^c^ | 7.4 ± 2.0  (88.4%)^b,c^ | 0.001 |
| **Day 14** | 8.5 ± 1.3  (86.8%) ^a^ | 7.8 ± 0.7  (87.8%) ^a^ | 2.9 ± 2.2  (95.4%)^b,c^ | 7.0± 2.1^c^  (89.5%) ^a,b^ | 5.5 ± 2.9  (91.4%)^a,b^ | 0.9 ± 0.3  (98.7%)^c^ | 5.0 ± 2.4^a,b^  (90.8%)^a,b^ | 0.000 |

Notes NO = no treatment (negative control), PCG = 15% positive control gamat, PCA = 0.1% positive control acriflavine, PE 0.1% = 0.1% polychaete extract, PE 0.5% = 0.5% polychaete extract, PE 1.0% = 1.0% polychaete extract prepared in cetomacrogol ointment, and NC = cetomacrogol ointment (negative control). Data were mean ± SEM. n = 5. The significance difference was analysed using 0ne-way ANOVA (*p <0.05) between 1.0% polychaete extract and negative control.

TABLE S3: Skin irritation score was recorded as ‘0’ according OECD guideline due to no oedema and erythema formation after rat’s skin was applied with different treatments for 1, 24, 48 and 72. Treatment utilized for skin irritation test were NC = cetomacrogol ointment (negative control), PE 0.1% = 0.1% polychaete extract, PE 0.5% = 0.5% polychaete extract and PE 1.0% = 1.0% polychaete extract prepared in cetomacrogol ointment.

| Skin reaction | Observation time (h) | Treatments | | | |
| --- | --- | --- | --- | --- | --- |
|  |  | NC | PE 0.1% | PE 0.5% | PE 1.0% |
| Oedema | 1 | 0 | 0 | 0 | 0 |
|  | 24 | 0 | 0 | 0 | 0 |
|  | 48 | 0 | 0 | 0 | 0 |
|  | 72 | 0 | 0 | 0 | 0 |
|  |  |  |  |  |  |
| Erythema | 1 | 0 | 0 | 0 | 0 |
|  | 24 | 0 | 0 | 0 | 0 |
|  | 48 | 0 | 0 | 0 | 0 |
|  | 72 | 0 | 0 | 0 | 0 |

TABLE S4: Hazardous materials in aqueous extract *Marphysa moribidii* emulsifying ointment (1.0% w/w). The result showed no detection of heavy metals such as arsenic, lead, cadmium and mercury in the polychaete ointment.

| **Heavy metals** | **Concentration (mg/kg)** | **NPRA limit (mg/kg)** |
| --- | --- | --- |
| Arsenic (As) | ND <0.1 | 5 |
| Cadmium (Cd) | ND <0.1 | 5 |
| Lead (Pb) | ND <0.1 | 20 |
| Mercury (Hg) | ND <0.01 | 1 |

ND = Not detected
